# RPL6: A Key Molecule Regulating Zinc- and Magnesium-Bound Metalloproteins of Parkinson’s Disease

**DOI:** 10.3389/fnins.2021.631892

**Published:** 2021-03-11

**Authors:** Athira Anirudhan, Paola Isabel Angulo-Bejarano, Prabu Paramasivam, Kalaivani Manokaran, S. Manjunath Kamath, Ram Murugesan, Ashutosh Sharma, Shiek S. S. J. Ahmed

**Affiliations:** ^1^Drug Discovery and Multi-omics Laboratory, Faculty of Allied Health Sciences, Chettinad Academy of Research and Education, Kelambakkam, India; ^2^School of Engineering and Sciences, Centre of Bioengineering, Tecnologico de Monterrey, Queretaro, Mexico; ^3^Department of Neurology, School of Medicine, University of New Mexico Health Sciences Center, University of New Mexico, Albuquerque, NM, United States; ^4^Department of Medical Laboratory Technology, Manipal College of Health Professions, Manipal, Manipal Academy of Higher Education, Manipal, India; ^5^Department of Pharmacology, Saveetha Dental College (SDC), Saveetha Institute of Medical and Technical Sciences, Chennai, India

**Keywords:** Parkinson’s disease, meta-analysis, metalloprotein network, gene expression, serum metals

## Abstract

Parkinson’s disease (PD) is a progressive neurodegenerative disease with no definite molecular markers for diagnosis. Metal exposure may alter cellular proteins that contribute to PD. Exploring the cross-talk between metal and its binding proteins in PD could reveal a new strategy for PD diagnosis. We performed a meta-analysis from different PD tissue microarray datasets to identify differentially expressed genes (DEGs) common to the blood and brain. Among common DEGs, we extracted 280 metalloprotein-encoding genes to construct protein networks describing the regulation of metalloproteins in the PD blood and brain. From the metalloprotein network, we identified three important functional hubs. Further analysis shows 60S ribosomal protein L6 (RPL6), a novel intermediary molecule connecting the three hubs of the metalloproteins network. Quantitative real-time PCR analysis showed that RPL6 was downregulated in PD peripheral blood mononuclear cell (PBMC) samples. Simultaneously, trace element analysis revealed altered serum zinc and magnesium concentrations in PD samples. The Pearson’s correlation analysis shows that serum zinc and magnesium regulate the RPL6 gene expression in PBMC. Thus, metal-regulating RPL6 acts as an intermediary molecule connecting the three hubs that are functionally associated with PD. Overall our study explores the understanding of metal-mediated pathogenesis in PD, which provides a serum metal environment regulating the cellular gene expression that may light toward metal and gene expression-based biomarkers for PD diagnosis.

## Introduction

Parkinson’s disease (PD) is one of the most prevalent neurodegenerative diseases occurring at the substantia nigra (SNc) region of the central nervous system (Lang and Lozano, 1998). Degeneration of neurons causes resting bradykinesia, postural instability, rigidity, and tremor. These symptoms, along with the Movement Disorder Society (MDS)-Unified Parkinson’s Disease Rating Scale (UPDRS), help in the clinical diagnosis of PD (Magrinelli et al., 2016). Imaging techniques such as CT, PET, and MRI showed benefit in PD detection (Pagano et al., 2016). Currently, there is no molecular method to diagnose PD and treatment to cure PD. The medications help in symptom management. Advances in research establish the underlying PD pathogenesis that allows determining molecular markers for diagnosis. Many studies implement high-throughput technologies, which generate vast biological data in genomics, transcriptomics, proteomics, and metabolomics. These biological resources publically available in repositories are useful for further investigation. Integrating these available resources may provide several previously unknown mechanisms of PD, which enables biomarker discovery.

Among biological data, meta-analysis of microarray-based gene expression profiles was extensively investigated to overcome the difficulties in conducting a large cohort study. Meta-analysis is a scientific method that systematically integrates the gene expression data of different populations on a similar entity to analyze and provide more reliable results (Ahn and Kang, 2018). Although several gene expression studies were conducted in PD, the potential limitation of obtaining the affected tissue restricts them to conduct an experiment in a larger sample size. A recent review highlights the restriction of less sample size in most PD gene expression studies (Kelly et al., 2019). Hence, a meta-analysis of combining the gene expression datasets of various PD studies will increase the sample size and provide more reliable results with a high statistical power. For instance, Chi et al. (2018) executed the meta-analysis and reported that mitogen-activated protein kinase 8 (MAPK8), cell division cycle 42 (CDC42), NADH:ubiquinone oxidoreductase core subunit S1 (NDUFS1), cytochrome C oxidase subunit 4I1 (COX4I1), and succinate dehydrogenase complex subunit C (SDHC) are the potential markers for PD diagnosis. However, Chi et al. (2018) conducted a meta-analysis in brain tissue, which diminishes the light to use these markers for clinical practices. Testing the genes that are pathologically connecting brain and blood through a meta-analysis will help develop blood-based biomarkers (Moni et al., 2019; Shen et al., 2019; Genoud et al., 2020). Such pathologically linked biomarkers will be more reliable and improve the diagnostic value.

Considering various environmental factors, metal exposure is one of the most frequently reported causative factors in PD. Several reports suggest altered metal concentration in PD (Forte et al., 2004; Bocca et al., 2006). However, the influence of metal in regulating PD genes in the context of biological network is limited. We hypothesized that the environmental metals alter the metal homeostasis that may influence the cellular metal-binding proteins to cause PD. In order to understand PD efficiently in the context of metal toxicity, the metalloprotein network involving metal-bound proteins and metals need to be studied.

## Materials and Methods

The retrieval, selection, analysis, and reporting of this study follow the Preferred Reporting Items for Systematic Reviews and Meta-Analyses (PRISMA) guidelines (Moher et al., 2015).

### Dataset Selection for Meta-Analysis

**FIGURE 1:**
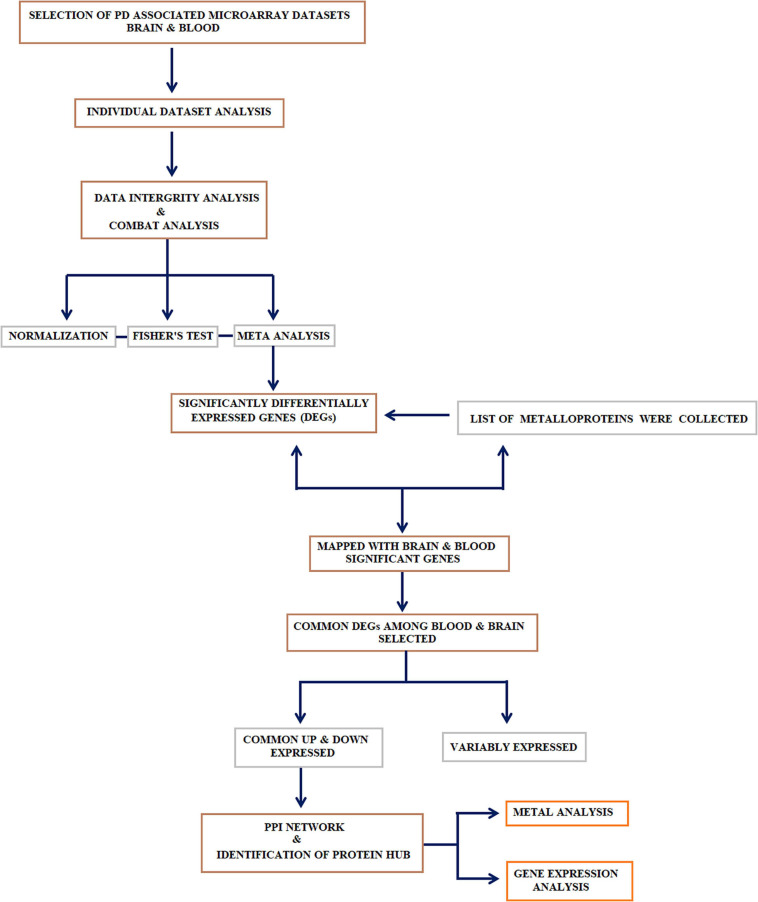
Study design: Systematic workflow describing the major steps adopted in this study: (1) collection, curation, and meta-analysis of datasets; (2) identification of differential genes and selecting the genes that encode metalloproteins; (3) genes that encode metalloproteins showing a common trend in the blood and brain were selected and subjected to metalloprotein network; (4) identification of the linker protein that connects the hubs extracted from the metalloprotein network; (5) recruitment of research participants to determine the gene expression linker protein in peripheral blood mononuclear cell (PBMC) and the serum metal concentration of proteins in the hubs.

Gene expression dataset searches were executed in NCBI Gene Expression Omnibus (GEO)^1^ and Array Express database^2^ by inception of database records between January 1990 and November 2018 using the keywords related to “Parkinson’s disease.” Two independent groups (SSJ, AS, and RM; PIA, PP, and MK) tested the procedure (Figure 1) of dataset search and retrieval following the inclusion and exclusion criteria as described. Inclusion criteria: (1) case-control study (*Homo sapiens*), (2) comparative studies between PD and other neurological diseases, (3) brain or whole blood as study specimens, (4) datasets include raw or processed data. Whereas the exclusion criteria include (1) animal model and *in vivo* studies, (2) secondary gene expression studies, (3) microarray dataset with less than two replicate samples in a group, and (4) studies other than microarray experiments.

### Data Extraction

Data extraction was performed independently by two authors (AA and KM) and cross-verified between them to eliminate errors. Any differences in opinion were resolved through group discussion. The information relating to each selected dataset, such as investigator name, year, accession number, sample source, array platform, number of samples (PD and control), expression data, study population, and primary and secondary outcome measures, was recorded. The characteristics of the dataset are included in Tables 1A,B.

**TABLE 1A:** The study characteristics of brain datasets included in the meta-analysis.

**Accession ID**	**Array platform number**	**Number of samples**	
		**Control**	**PD**
GSE7621	GPL570 [HG-U133_Plus_2]	9	16
GSE8397	GPL96 [HG-U133A]	19	28
	GPL97 [HG-U133B]	17	29
GSE19587	GPL571 [HG-U133A_2]	10	12
GSE20141	GPL570 [HG-U133_Plus_2]	8	10
GSE20146	GPL570 [HG-U133_Plus_2]	10	10
GSE20163	GPL96 [HG-U133A]	9	8
GSE20164	GPL96 [HG-U133A]	5	6
GSE20168	GPL96 [HG-U133A]	15	14
GSE20186	GPL96 [HG-U133A]	14	14
	GPL6947 Illumina HumanHT-12 V3.0 expression beadchip	17	16
GSE20291	GPL96 [HG-U133A]	20	15
GSE20292	GPL96 [HG-U133A]	18	11
GSE20295	GPL96 [HG-U133A]	53	40
GSE28894	GPL6104 Illumina humanRef-8 v2.0 expression beadchip	59	55
GSE49036	GPL6104 Illumina humanRef-8 v2.0 expression beadchip	8	20
GSE42966	GPL4133 Agilent-014850 Whole Human Genome Microarray 4 × 44K G4112F	6	9

**PD, Parkinson’s disease.**

**TABLE 1B:** The study characteristics of blood datasets included in the meta-analysis.

**Accession ID**	**Array platform number**	**Number of samples**	
		**Control**	**PD**
GSE6613	GPL96 [HG-U133A]	22	50
GSE22491	GPL6480 Agilent-014850 Whole Human Genome Microarray 4 × 44K G4112F	8	10
GSE54536	GPL10558 Illumina HumanHT-12 V4.0 expression beadchip	4	4
GSE72267	GPL571 [HG-U133A_2]	19	40
GSE18838	GPL5175 [HuEx-1_0-st] Affymetrix Human Exon 1.0 ST Array [transcript (gene) version]	12	18
GSE49126	GPL4133 Agilent-014850 Whole Human Genome Microarray 4 × 44K G4112F	20	30
GSE57475	GPL6947 Illumina HumanHT-12 V3.0 expression beadchip	49	93
GSE99039	GPL570 [HG-U133_Plus_2]	234	239

### Analysis of Individual Dataset

Each dataset was background corrected and normalized using Robust Multi-chip Averaging (RMA) method. Principal component analysis (PCA) plot was visualized to determine the sample distribution based on gene expression before and after normalization to identify outliers. The differentially expressed genes (DEGs) in PD compared to control was computed using the limma method (Derkow et al., 2018) with *p*-value < 0.05, adjusted by Benjamini–Hochberg’s false discovery rate (FDR).

### Meta-Analysis

Meta-analysis was performed using MetaDE package in R (Wang et al., 2012) by following the procedure of Su et al. (2019). Datasets from the same tissue (blood or brain) were combined to perform the meta-analysis. The RMA processed expression datasets were then merged by converting probe ID of each gene to Entrez ID, which allows comparing the same gene across different datasets. Further, the homogeneity of all the collected datasets was verified using MetaDE package in R (Wang et al., 2012). Further, Fisher’s method was used with *p*-value < 0.05 from the multiple datasets to determine the significantly differentially expressed meta-analyzed PD genes of the brain and blood.

### Metalloproteins and Differential Expression

Next, the metal-bound protein (93 metals of the periodic table) search was executed in Protein Data Bank (PDB) by applying the search filters that include: (1) Organism type: *Homo sapiens*; (2) Experimental method: XRD; (3) Molecule type: Protein; (4) Sequence feature: Wild-type protein; and (5) X-ray resolution: > 3A°. Overall, we retrieved 6,367 proteins that bound with 60 different metals. Each metalloprotein was named using its official gene symbol. Among 6,367 metalloprotein-encoding genes, we only took DEGs that are commonly overexpressed and downregulated in both blood and brain of PD. This exercise yielded 280 DEGs, which served input for metalloprotein network construction.

### Metalloprotein Network Construction, Cluster Identification, and Pathway Enrichment

Cytoscape v3.1 is a network biology software that enables to construct and investigate the complex interaction of genes and proteins using a BisoGene plug-in. The BisoGene retrieves protein–protein interaction (PPI) data from the databases DIP, BIOGRID, HPRD, BIND, MINT, and INTACT to create the network (metalloprotein network). The 280 metalloproteins were given as input to create interaction networks connecting each other’s edges. Among 280 metalloproteins, 90 forms the protein network. Whereas the other proteins were noticed to have a maximum of one interacting partner and few were without interaction. The constructed network was visualized using Cytoscape network visualizer, which shows the interconnectivity among dysregulating metalloproteins. Additionally, the topological characteristics of the network were tested using the network analyzer plug-in of Cytoscape. From the network, we extracted the hubs that are highly connected using the Clustering with Overlapping Neighborhood Expansion (ClusterONE) algorithm (Nepusz et al., 2012). ClusterONE detects the hub from overlapping protein complexes of the PPI data and ranked them based on *p*-value. The top three hubs showing the highest significant *p*-values were selected and subjected to Kyoto Encyclopedia of Genes and Genomes (KEGG) database for pathway analysis. Simultaneously, the hub proteins and their connections were investigated to identify any intermediary molecules connecting the three hubs. The gene expression of intermediary molecule was analyzed in PD. Also, the concentrations of the metals that bound to the hub proteins were examined in PD serum compared to healthy control.

### Experimental Validation in Parkinson’s Disease Samples

We obtained samples from PD patients (PD = 33) who underwent clinical examination and MDS-UPDRS grading by a movement disorder specialist at the Chettinad Hospital and Research Institute, Tamil Nadu, India (Table 2). Similarly, the participants following the below criteria served as the control sample (control = 32). Our inclusion criteria were: (1) aged group between 55 and 75 years and (2) body mass index (BMI) of 18–25 kg/m^2^. Whereas the exclusion criteria include (1) previous history of alcohol abuse; (2) smoker or tobacco user; (3) any severe systemic diseases; (4) participants under minerals and chelating agents as supplements; (5) participants who underwent any surgery in the last 8 months; and (6) presence of any secondary parkinsonism due to accident, trauma, or drugs. Before sample collection, the signed informed consent was obtained from all the participants. Peripheral blood 6 ml (3 ml + 3 ml) was collected in the Vacutainer tube (BD, United States). Then, 3 ml blood was centrifuged immediately for 10 min at 3,500 rpm, and we stored serum at −80°C for metal analysis. We used the remaining 3 ml for the isolation of peripheral blood mononuclear cell (PBMC) and RNA extraction for the gene expression analysis.

**TABLE 2:** Clinical characteristics and metal concentrations in serum determined by AAS showing significant changes in magnesium and zinc in PD compared with control.

**Metals**	**Control (*n* = 32)**	**PD (*n* = 33)**	***p*-value**
Age (years)	65.21 ± 5.60	66.21 ± 5.41	0.48
Gender	Male: 15; Female: 7	Male: 14; Female: 9	0.84
Age at onset (years)	−	63.84 ± 5.4	−
UPDRS I–Mentation, Behavior, and Mood	−	2.93 ± 2.10	−
UPDRS II–Activities of Daily Living (ADL)	−	11.34 ± 7.31	−
UPDRS III–Motor Examination	−	21.6 ± 18.22	−
**Serum (μg/L)**			
Magnesium	4.31 ± 0.041	5.316 ± 0.039	≤0.001*
Zinc	2.846 ± 0.046	2.631 ± 0.039	≤0.001*

*p < 0.05, statistical significance; mean ± SD. AAS, atomic absorption spectrophotometer; PD, Parkinson’s disease; UPDRS, Unified Parkinson’s Disease Rating Scale.

### Serum Metal Analysis

All precautions were made by following the National Committee for Clinical Laboratory Standards (NCCLS) guidelines to avoid contamination throughout the sample processing of metal estimation. The nitric acid-based microwave digestion was carried out to extract the metals from the serum. Flame atomic absorption spectrophotometer (AAS) (Tokiya and Toda, 1977) was used to determine the serum zinc (Zn) and magnesium (Mg) concentration. For the calibration, NIST SRM 3100 series single-element standard solution was used at various concentrations to have a standard graph. Also, the blank solution was used to determine the limits of detection (LoDs) for the Zn and Mg.

### Gene Expression Analysis

PBMCs were isolated using Histopaque-1077 (Sigma-Aldrich) following the manufacturer’s protocol. RNA was extracted from the PBMC using TRIzol (Invitrogen) reagent and quantified using Nanodrop 2000 (Thermo Fisher Scientific). The 60S ribosomal protein L6 (RPL6) gene expression was examined in PD and control using quantitative real-time PCR (ABI-7000, Applied Biosystems) using forward (GACGGGAATGAGAAAGGCCA) and reverse (AGCCCTGGAGCTGAGGAATA) primers. We used the gene GAPDH as internal control (forward: AAGGTGAAGGTCGGAGTCAA and reverse: ACATGTAAACCATGTAGTTGAGGT). Finally, the relative expression was measured following the 2^–ΔΔ*Ct*^ method.

### Statistical Analysis

All data were tested for normal distribution. Further, the statistical significance (*p* < 0.05) between PD and control was assessed using the Student’s *t*-test method. The interdependency between metal concentration and RPL6 gene expression was calculated by Pearson’s correlation method. All statistical analyses were carried using the SPSS version 21 software.

## Results

### Dataset Collection

We used keywords related to PD to search for the gene expression studies conducted in the brain and blood in the databases. The datasets following our inclusion and exclusion criteria were selected to have 15 datasets from the brain (GSE7621, GSE8397, GSE19587, GSE20141, GSE20146, GSE20163, GSE20164, GSE20168, GSE20186, GSE20291, GSE20292, GSE20295, GSE28894, GSE49036, and GSE42966) containing 610 samples (control = 297 and PD = 313) and eight datasets from the blood tissue (GSE6613, GSE22491, GSE54536, GSE72267, GSE18838, GSE49126, GSE57475, and GSE99039) containing 852 samples (control = 368, PD = 484). The detailed characteristics of the datasets included in this study are presented in Tables 1A,B.

### Meta-Analysis of the Blood and Brain Datasets

Prior to the meta-analysis, each dataset was RMA processed and DEGs between PD and control were determined using the limma package. To identify common DEGs in the blood and brain between PD and control samples, the datasets from the blood and brain were meta-analyzed with the *p* < 0.05 as cutoff in Fisher’s exact test. Overall, 1,951 DEGs in the blood and 3,984 DEGs in the brain were obtained. Simultaneously, 3,453 metal-bound proteins were collected from the PDB, as described in the “Materials and Methods” section. We converted each metal-bound protein into the official gene symbol and mapped with 1,951 (blood) and 3,984 (brain) differentially expressed meta-analyzed PD genes. A total of 280 DEGs were identified in PD showing common trends in both tissues. These 280 DEGs were used for the network construction.

### Metalloprotein Network and Pathway Analysis

**FIGURE 2:**
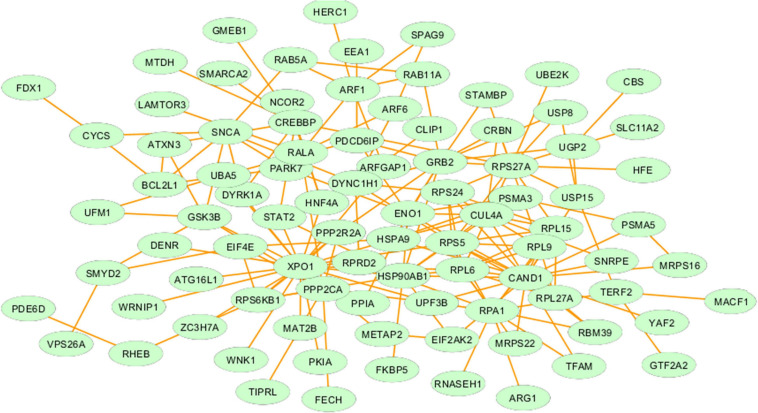
Metalloprotein network representing the interaction between metalloproteins that are commonly upregulated and downregulated in both the brain and blood of Parkinson’s disease patients.

**FIGURE 3:**
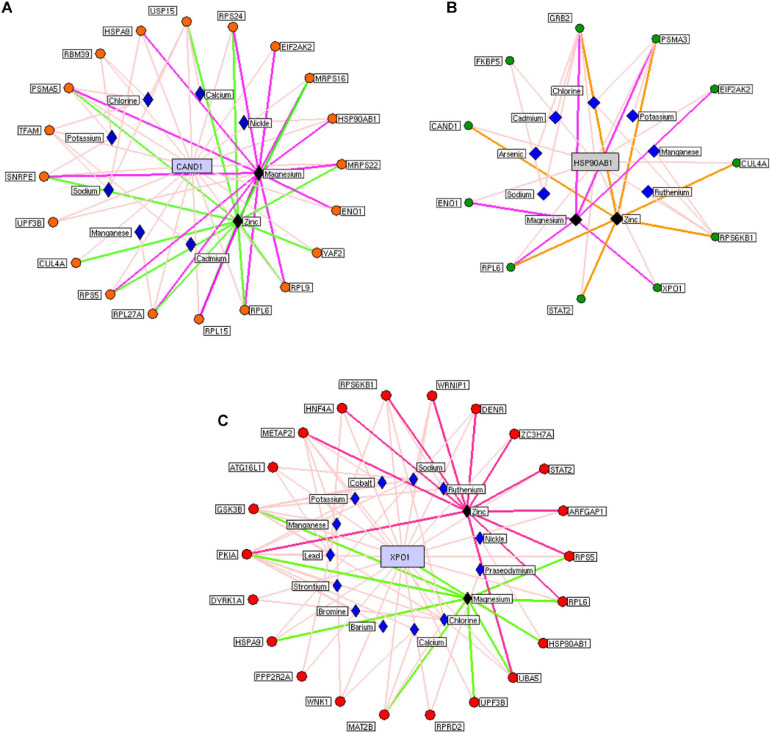
Highly interactive metalloprotein hubs: Three highly interconnected protein hubs (**A–C**) extracted from the metalloprotein network. Colored circular nodes represent metalloproteins connecting to the core protein (blue rectangular box). The edges (colored line) between the proteins and their cofactor metals (violet rhombus). Black rhombus representing the cofactors that bound with more metalloproteins in the hubs.

**FIGURE 4:**
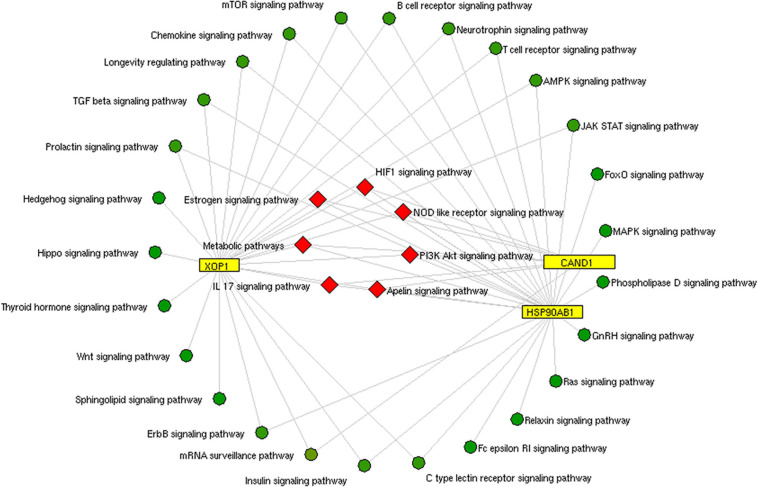
Pathway enrichment of hubs: The common pathway (red rhombus) shared by three protein hubs (yellow rectangle) extracted from the metalloprotein network.

**FIGURE 5:**
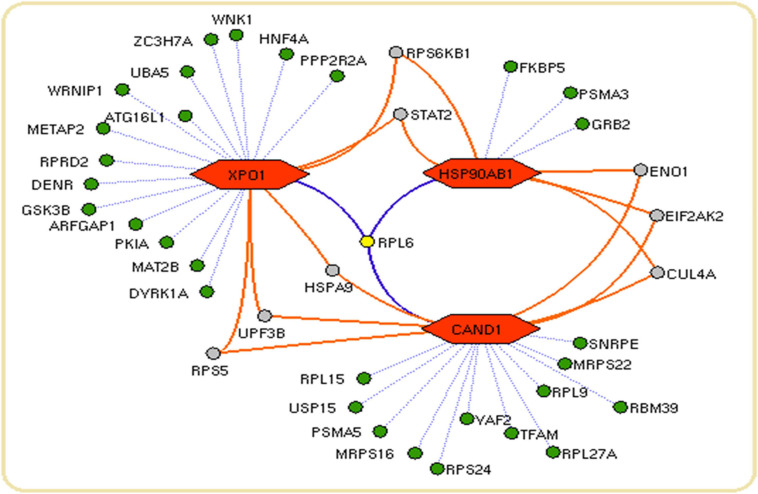
Network connectivity of hub proteins: The network showing the proteins (green nodes) with their connected interaction (dotted violet edges). The 60S ribosomal protein L6 (RPL6) (yellow node) with violet edges that connects core proteins (orange hexagon) of the hubs. The protein (gray node) connects a maximum of two core proteins (orange hexagon) in the network.

We constructed the metalloprotein network using Cytoscape v3.10. Among 280 metalloproteins, 90 forms the protein network that explicates 177 interconnected edges (Figure 2). Whereas the other proteins were noticed with a maximum of one interacting partner and few were without an interaction partner. The topological characteristics were assessed for the network (Supplementary Table S1). We used ClusterONE algorithm to identify the top 3 important hubs that are ranked based on *p*-values. Hub1 contains XPO1 with 22 interacting proteins, hub2 contains CAND1 with 20 connecting proteins, and hub3 contains HSP90AB1 connected with 11 proteins (Figure 3 and Supplementary Table S2). Each hub was subjected to pathway enrichment, which showed an association with 61 molecular pathways (XPO1 hub was associated with 26 pathways, CAND1 hub linked with eight pathways, and HSP90AB1 hub was related to 27 pathways) using the KEGG database. Among 61 molecular pathways, seven were noticed common to all three hubs (Figure 4). Further, investigation using Cytoscape network analyst plug-in shows involvement of 42 proteins in the hubs, and few were common among them. Particularly, RPL6 acts as a key linker protein that connects all the three hubs (Figure 5). Subsequently, metal cofactor for the proteins in the hubs was identified (Figure 3), which shows that Mg and Zn are the cofactors for most of the metalloproteins in the hubs. Interestingly, Mg and Zn were noticed bound with RPL6 proteins (PDB ID: 6T59).

### Serum Metal and Peripheral Blood Mononuclear Cell Gene Expression

**FIGURE 6:**
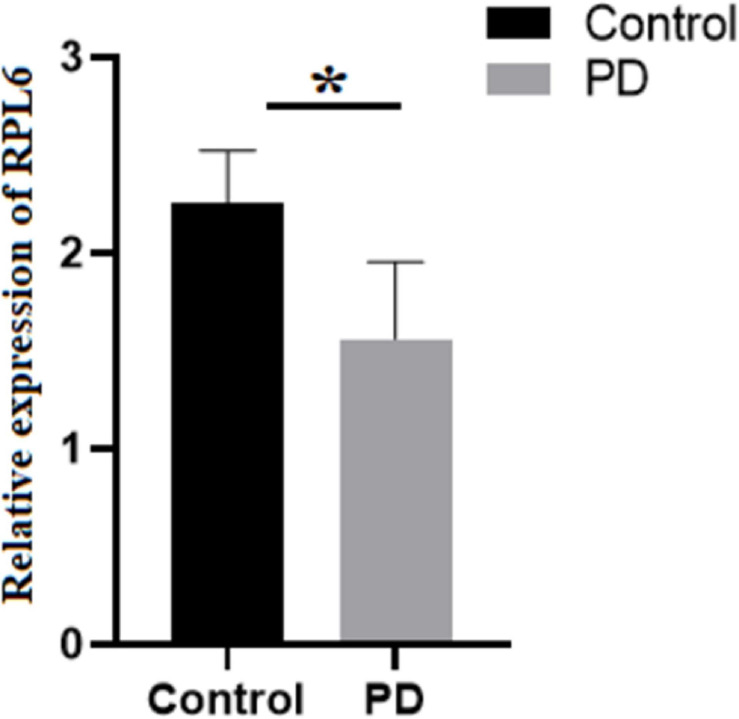
Relative expression: Gene expression analysis of 60S ribosomal protein L6 (RPL6) in peripheral blood mononuclear cells (PBMCs) of control (black) and PD (gray). The columns represent the average gene expression with standard deviation error bar, and asterisk denotes statistical significance with *p*-value ≤ 0.05.

The levels of Mg and Zn were assessed in the blood serum of PD and control using AAS. The normality test was conducted, which showed normal distribution of data. Further, Student’s test was executed that demonstrated that Mg was significantly increased, whereas Zn was significantly decreased (Table 2) in PD compared to control. Similarly, the gene expression of RPL6 was decreased significantly (*p* < 0.05) in PD (Figure 6). Furthermore, the interdependency between metal concentrations and gene expression shows RPL6 was negatively correlated with Mg and positively correlated with Zn (Table 3) based on R > 0.90.

**TABLE 3:** Pearson’s correlation between serum metal concentrations with the RPL6 gene expression showing interdependency in Parkinson’s disease.

**Metal**	**Gene**	**R**	**Statistical significance (*p*-value)**
Magnesium	RPL6	−0.99	0.02*
Zinc		0.99	0.03*

*p < 0.05, statistical significance. RPL6, 60S ribosomal protein L6.

## Discussion

We performed a meta-analysis to identify common DEGs in PD brain and blood that allows new biological insights in PD pathogenesis. To our knowledge, this is one of the largest gene expression meta-analyses conducted in PD using 15 microarray gene expression datasets of the brain containing 610 samples and eight datasets in whole blood containing 852 samples to overcome the limitation expressed by Kelly et al. (2019). Currently, there is no well-defined molecular marker available for PD diagnosis; still the MDS-UPDRS, a gold standard method, is followed in clinical setup. A recent advancement in multi-omics technologies enables to identify biomarkers in body fluids for complex diseases (Olivier et al., 2019).

In the past decades, progression in PD research has been shown to be exponentially productive; however, identifying the influence of cellular metal on causative genes in PD pathogenesis is needed. Therefore, we attempted to link the serum metal concentration with the DEGs of PD. We identified DEGs by meta-analysis by merging multiple PD-associated gene expression datasets. This resulted in 280 DEGs from a protein network containing 90 proteins with 177 edges contributing to three important PD hubs representing molecular pathways.

Insulin signaling pathway is one of the molecular pathways represented by the hubs (Figure 4). Several studies reported the impaired insulin signaling as a risk factor for PD (Bosco et al., 2012; Pang et al., 2016). Particularly, Bosco et al. (2012) observed a change in insulin mechanism in the cortex and the limbic PD system. Recently, Fiory et al. (2019) showed an altered blood–brain barrier (BBB) integrity in the striatum region that causes a change in the insulin signaling mechanism. Also, Khang et al. (2015) show that the insulin resistance dysregulates mitochondrial proteins at the substantia nigra (Moreira et al., 2006; Duarte et al., 2012). The disrupted mitochondrial function generates an excess of reactive oxygen species (ROS) that causes neuronal death (Dias et al., 2013). Changes in insulin signaling and mitochondrial dysfunction accelerate degeneration of dopaminergic neurons (Kleinridders et al., 2015). Also, the analysis of hubs showed involvement of mammalian target of rapamycin (mTOR) signaling and ErbB signaling pathways in PD pathogenesis. Dysregulated mTOR signaling was reported by Xu et al. (2014) in PD. Interestingly, Bockaert and Marin (2015) and Dijkstra et al. (2015) illustrate the upregulation of mTOR signaling pathway that accumulates α-synuclein in stages 1 and 2 of PD.

On the other hand, ErbB signaling plays a vital role in the central nervous system. Dysregulated ErbB signaling was noticed in our pathway analysis of hubs. In PD, Iwakura et al. (2005); Iwakura and Nawa (2013) report the downregulation of ErbB that causes degeneration of dopaminergic neurons at the midbrain region. Additionally, the ErbB takes part in controlling the glutamatergic function at hippocampus and midbrain dopaminergic neurons that are associated with cognitive and behavior functions (Ledonne et al., 2018). Recently, Ledonne and Mercuri (2020) suggest that the neuregulin (NRG)/ErbB-dependent synaptic plasticity at the hippocampus contributes to the memory process. The ErbB4 activation in dopaminergic neurons inhibits dopamine transporter (DAT) that increases the level of extracellular dopamine. Thus, frequent activation of dopamine (D4) receptor on hippocampus CA1 pyramidal neurons alters the long-term potentiation (LTP) induction or expression (Ledonne and Mercuri, 2020). Though we identified several molecular pathways, we tried to explain the importance of our study by describing the insulin, mTOR, and ErbB signaling pathways in PD.

Subsequent analysis of hubs showed that most hub proteins were bound with Zn and Mg (Figure 3). Also, emerging evidence suggests that these pathways may be regulated by Zn and Mg. Studies show Zn involvement in regulating the insulin pathway to maintain the homeostasis of glucose (Fukunaka and Fujitani, 2018; Norouzi et al., 2018). Zn takes part in ErbB signaling for cellular growth and differentiation (Lund et al., 2005). Szewczyk et al. (2015) suggest that decreased Zn alters the phosphorylation of mTOR signaling that dysregulates synapse and synaptic proteins. Importantly, the altered Zn homeostasis causes a change in behavior, memory, learning, and emotional stability (Takeda, 2000). Meta-analysis of Ke Du et al. (2017) reports decreased circulating Zn in PD patients, which is in agreement with our result. Similarly, Mg plays a significant role in neuromuscular signal conduction, energy synthesis, and releasing neurotransmitters (Oyanagi et al., 2006). Significant increase in Mg was noticed in our study; a similar result was noticed in the meta-analysis of Jin et al. (2018). Additionally, this result was further supported by our previous study (Ahmed and Santosh, 2010). Mg is essential for maintaining glucose metabolism and insulin mechanism (Kostov, 2019). Also, Mg promotes mTOR signaling (Lu et al., 2018), which confirms metals contributing to PD pathogenesis. Additionally, investigation of hub proteins showed RPL6 is the key linker protein that connects all three hubs. RPL6 encodes 60S ribosomal subunit that plays a vital role in oxidative phosphorylation, synaptic transmission, and neuronal signaling in PD (Hamed et al., 2018). Recently, Duarte et al. (2019) suggested that downregulation of RPL6 activates AMPK signaling in PD. Similarly, downregulation of RPL6 in PBMC was observed in PD compared to control (Figure 6). Interestingly, Hamed et al. (2018) report downregulation of RPL6 in early onset, which supports early detection of PD. Also, our result suggests that altered metal homeostasis might cause a change in RPL6 gene expression, which supports our hypothesis of metal regulating cellular gene expression causing PD. Overall, our study elucidates the complex interconnection between metals and metalloproteins in PD pathogenesis. However, there are limitations in our study: (1) we have not analyzed the expression of all interacting proteins in the hubs of the metalloprotein network, (2) the number of samples enrolled for the study is limited, and (3) the participants involved in this study were south Indian population. On the other hand, it is important to acknowledge the advantages of this study: (1) this is one of the largest meta-analyses conducted in PD based on brain and blood gene expression profiles, (2) this study uses a novel approach that integrates the multi-omics data to provide a wider view on pathogenesis, and (3) this study provides substantial experimental evidence for the interplay between metals and cellular gene expression in PD.

## Conclusion

In conclusion, to our knowledge, this is the first meta-analysis that investigates the association of metalloproteins and metals in PD. Our study confirms the change in serum Zn and Mg concentration in PD compared to control. Also, the altered metals contribute change in gene expression of key linker proteins connecting PD-associated hubs. Together, relating the gene expression with metal concentration can be used as biomarkers for PD diagnosis.

## Data Availability Statement

Publicly available datasets were analyzed in this study. This data can be found here: https://www.ncbi.nlm.nih.gov/geo/query/acc.cgi?acc=GSE8397

https://www.ncbi.nlm.nih.gov/geo/query/acc.cgi?acc~=~GSE19587

https://www.ncbi.nlm.nih.gov/geo/query/acc.cgi?acc~=~GSE20141

https://www.ncbi.nlm.nih.gov/geo/query/acc.cgi?acc~=~GSE20146

https://www.ncbi.nlm.nih.gov/geo/query/acc.cgi?acc~=~GSE20163

https://www.ncbi.nlm.nih.gov/geo/query/acc.cgi?acc~=~GSE20164

https://www.ncbi.nlm.nih.gov/geo/query/acc.cgi?acc~=~GSE20168

https://www.ncbi.nlm.nih.gov/geo/query/acc.cgi?acc~=~GSE20186

https://www.ncbi.nlm.nih.gov/geo/query/acc.cgi?acc~=~GSE20291

https://www.ncbi.nlm.nih.gov/geo/query/acc.cgi?acc~=~GSE20292

https://www.ncbi.nlm.nih.gov/geo/query/acc.cgi?acc~=~GSE20295

https://www.ncbi.nlm.nih.gov/geo/query/acc.cgi?acc~=~GSE28894

https://www.ncbi.nlm.nih.gov/geo/query/acc.cgi?acc~=~GSE49036

https://www.ncbi.nlm.nih.gov/geo/query/acc.cgi?acc~=~GSE42966

https://www.ncbi.nlm.nih.gov/geo/query/acc.cgi?acc~=~GSE6613

https://www.ncbi.nlm.nih.gov/geo/query/acc.cgi?acc~=~GSE22491

https://www.ncbi.nlm.nih.gov/geo/query/acc.cgi?acc~=~GSE54536

https://www.ncbi.nlm.nih.gov/geo/query/acc.cgi?acc~=~GSE72267

https://www.ncbi.nlm.nih.gov/geo/query/acc.cgi?acc~=~GSE18838

https://www.ncbi.nlm.nih.gov/geo/query/acc.cgi?acc~=~GSE49126

https://www.ncbi.nlm.nih.gov/geo/query/acc.cgi?acc~=~GSE57475

https://www.ncbi.nlm.nih.gov/geo/query/acc.cgi?acc~=~GSE99039.

## Ethics Statement

The studies involving human participants were reviewed and approved by the Institutional Human Ethical Committee, Chettinad Academy of Research and Education. The patients/participants provided their written informed consent to participate in this study.

## Author Contributions

AA and PA-B conducted the experimental work and analysis and interpretation of the data. PP supervised the gene expression analysis. SA and AS designed the study, performed part of the experiment and the analysis and interpretation of the data, and critically revised the manuscript. RM supervised the statistical analysis. SK and KM provided critical suggestion in the design of the experiment and manuscript correction. Particularly, SA, RM, PP, and SK were involved in validating the procedure for dataset collection for meta-analysis. All authors read and approved the current version of the manuscript.

## Conflict of Interest

The authors declare that the research was conducted in the absence of any commercial or financial relationships that could be construed as a potential conflict of interest.

## Footnotes

1 http://www.ncbi.nlm.nih.gov/geo/

2 http://www.ebi.ac.uk/arrayexpress/

## Supplementary Material

Supplementary Table 1 Topological characteristic of the network.

Supplementary Table 2 Average fold change in PD of merged dataset and combined *p*-values of the genes in the hubs.
