# Supplementary material for: RPL6: A Key Molecule Regulating Zinc- and Magnesium-Bound Metalloproteins of Parkinson’s Disease
Source: Front Neurosci. 2021 Mar 11;15:631892. doi: 10.3389/fnins.2021.631892 (PMC8006920; doi:10.3389/fnins.2021.631892)
Supplement: Supplementary Table 1 — Topological characteristic of the network. [file Data_Sheet_1.docx]

**Supplementary Table S1.** Topological characteristic of the network

| **Network Topologies** | **Score** |
| --- | --- |
| Clustering coefficient | 0.128 |
| Connected components | 34 |
| Network diameter | 7 |
| Network radius | 4 |
| Network centralization | 0.159 |
| Shortest path | 8010 (53%) |
| Characteristic path length | 3.300 |
| Avg. No.of neighbours | 2.878 |
| Network density | 0.024 |
| Network heterogeneity | 1.324 |

**Supplementary Table S2**. Average fold change in PD of merged dataset and combined p-values of the genes in the hubs.

| **Gene Name** | **BRAIN** | | **BLOOD** | |
| --- | --- | --- | --- | --- |
|  | **Average fold change in PD of merged datasets** | **Combined P- value** | **Average fold change in PD of merged datasets** | **Combined P- value** |
| ARFGAP1 | -0.400447088 | 8.85E-06 | -0.575722207 | 1.63E-02 |
| ATG16L1 | -0.591436811 | 4.89E-09 | -0.475284208 | 4.21E-02 |
| CAND1 | -1.206164407 | 4.41E-09 | -0.284103965 | 2.40E-02 |
| CUL4A | -1.306164407 | 8.92E-05 | -0.792411688 | 4.20E-03 |
| DENR | -1.199140393 | 1.18E-12 | -0.448614756 | 2.92E-04 |
| DYRK1A | + 3.647383548 | 3.34E-09 | + 2.647383548 | 2.31E-04 |
| EIF2AK2 | -1.479639289 | 6.25E-08 | -0.484286133 | 1.64E-02 |
| ENO1 | -1.76835585 | 0.002795 | -0.702064364 | 3.26E-03 |
| FKBP5 | -0.419203906 | 1.3E-05 | -0.584202934 | 2.94E-02 |
| GRB2 | -1.374258561 | 0.002753 | -0.671213267 | 4.27E-02 |
| GSK3B | -0.248943505 | 3.86E-06 | -0.365492131 | 1.63E-02 |
| HNF4A | + 2.158028043 | 0.001694 | + 0. 4912112 | 2.88E-03 |
| HSP90AB1 | -3.001274952 | 0.001083 | -0.338908057 | 5.16E-03 |
| HSPA9 | -1.668379821 | 0.004195 | -0.459678577 | 2.62E-03 |
| MAT2B | -0.866365172 | 8.39E-10 | -0.568543851 | 6.19E-03 |
| METAP2 | -1.803167206 | 1.55E-06 | -0.745626383 | 4.06E-02 |
| MRPS16 | -0.459251417 | 0.034939 | -0.550398761 | 1.15E-02 |
| MRPS22 | -2.156832852 | 4.24E-05 | -0.553335105 | 4.28E-02 |
| PKIA | -0.177659362 | 3.5E-09 | -0.676341261 | 4.31E-02 |
| PPP2R2A | -0.818477548 | 0.000732 | -0.297575268 | 3.98E-02 |
| PSMA3 | -0.914882494 | 3.47E-05 | -0.699379163 | 5.17E-04 |
| PSMA5 | -0.198863964 | 1.44E-05 | -0.677987537 | 8.88E-03 |
| RBM39 | -0.699379163 | 0.004343 | -0.544762502 | 1.62E-02 |
| RPL15 | -2.279321542 | 5.88E-07 | -0.767797349 | 3.16E-02 |
| RPL27A | -1.235353734 | 0.000655 | -0.81269289 | 1.29E-02 |
| **RPL6** | **-1.634400711** | **0.001956** | **-0.73388349** | **2.42E-02** |
| RPL9 | -1.355519753 | 0.001004 | -0.802927776 | 2.67E-03 |
| RPRD2 | -1.803167206 | 5.96E-10 | -0.483258878 | 2.01E-02 |
| RPS24 | -0.602601071 | 5.13E-05 | -0.914086537 | 3.49E-02 |
| RPS5 | -3.852801984 | 2.4E-05 | -0.54149594 | 1.83E-02 |
| RPS6KB1 | -0.42785558 | 4.14E-10 | -0.494995701 | 2.62E-03 |
| SNRPE | -0.602601071 | 0.000382 | -0.631157072 | 1.04E-03 |
| STAT2 | -0.707212585 | 0.000441 | -0.397119497 | 2.75E-02 |
| TFAM | -0.016411023 | 0.000226 | -0.587452631 | 1.92E-03 |
| UBA5 | -0.311627584 | 1.93E-07 | -0.547646432 | 9.66E-03 |
| UPF3B | -0.77349582 | 5.73E-08 | -0.40750978 | 3.17E-04 |
| USP15 | -0.311456029 | 1.84E-05 | -0.363943846 | 3.15E-02 |
| WNK1 | -0.671526064 | 9.09E-05 | -0.433279562 | 5.94E-03 |
| WRNIP1 | -0.016411023 | 3.16E-09 | -0.655472974 | 4.18E-02 |
| XPO1 | -0.72238593 | 1.17E-05 | -0.342452633 | 4.11E-02 |
| YAF2 | -0.821046757 | 9.35E-09 | -0.639715721 | 2.49E-03 |
| ZC3H7A | -0.487983172 | 0.000236 | -0.522927011 | 6.60E-03 |

* p=value < 0.05 significant, negative sign represent down-regulated ; positive sign represent up-regulation .
